# Construction of Ancestral Chromosomes in Gymnosperms and the Application in Comparative Genomic Analysis

**DOI:** 10.3390/plants14152361

**Published:** 2025-08-01

**Authors:** Haoran Liao, Lianghui Zhong, Yujie He, Jie He, Yuhan Wu, Ying Guo, Lina Mei, Guibing Wang, Fuliang Cao, Fangfang Fu, Liangjiao Xue

**Affiliations:** State Key Laboratory of Tree Genetics and Breeding, Co-Innovation Center for Sustainable Forestry in Southern China, Key Laboratory of Forest Genetics and Biotechnology of Ministry of Education, Nanjing Forestry University, Nanjing 210037, China; haoranliao@njfu.edu.cn (H.L.); heyuie@njfu.edu.cn (Y.H.); yuhanwu1993@163.com (Y.W.); fffu@njfu.edu.cn (F.F.)

**Keywords:** gymnosperm, ancestral karyotype, collinearity, chromosome stability, LINE-1

## Abstract

Chromosome rearrangements during plant evolution can lead to alterations in genome structure and gene function, thereby influencing species adaptation and evolutionary processes. Gymnosperms, as an ancient group of plants, offer valuable insights into the morphological, physiological, and ecological characteristics of early terrestrial flora. The reconstruction of ancestral karyotypes in gymnosperms may provide critical clues for understanding their evolutionary history. In this study, we inferred the ancestral gymnosperm karyotype (AGK), which comprises 12 chromosomes, and conducted a collinearity analysis with existing gymnosperm genomes. Our findings indicate that chromosome numbers have remained remarkably stable throughout the evolution of gymnosperms. For species with multiplied chromosome numbers, such as gnetophytes, weak collinearities with the AGK were observed. Comparisons between the AGK and gnetophyte genomes revealed a biased pattern regarding retained duplication blocks. Furthermore, our analysis of transposable elements in *Welwitschia mirabilis* identified enriched regions containing LINE-1 retrotransposons within the syntenic blocks. Syntenic analysis between the AGK and angiosperms also demonstrated a biased distribution across chromosomes. These results provide a fundamental resource for further characterization of chromosomal evolution in gymnosperms.

## 1. Introduction

Chromosome evolution, which includes chromosomal rearrangement, polyploidization, and subsequent diploidization following polyploidization events, plays a significant role in driving species differentiation, adaptation, and diversification [1]. The evolutionary process results in observable changes in the karyotype that influence the chromosome number, size, and morphological characteristics of a species. The evolution of karyotypes illustrates the progressive transformation of chromosomal arrangements from ancestral genomes to contemporary forms. Consequently, karyotype analysis serves as an essential tool for investigating chromosome inversions, translocations, and deletions. It provides a fundamental framework for studying species relationships, chromosome counts, and structural variations.

Many efforts have been made to reconstruct ancestral karyotypes using bioinformatics methods to study species evolution. Li et al. revealed extensive paleohybridization events during early divergence by reconstructing the ancestral core *Brassicaceae* karyotype, demonstrating a conserved genomic architecture with traces of ancient hybridization [2]. The reconstruction of both the ancestral eudicot karyotype and ancestral core eudicot karyotype demonstrates that modern oak chromosomes evolved through successive chromosomal fusions, arm exchanges, and rearrangements.

This karyotype reorganization is coordinated with lineage-specific long terminal repeat retrotransposons (LTR-RTs) that jointly drive adaptive evolution [3]. Recent studies suggest that the ancestral grass karyotype has remained highly conserved in subfamilies such as *Bambusoideae* and *Oryzoideae*. However, lineage-specific chromosomal rearrangements emerging post-BOP (*Bambusoideae*, *Oryzoideae*, and *Pooideae*)-PACMAD (*Panicoideae*, *Aristidoideae*, *Chloridoideae*, *Micrairoideae*, *Arundiaceae*, and *Danthonioideae*) divergence are proposed as pivotal evolutionary mechanisms driving diversification within *Poaceae* [4]. Additionally, ancestral karyotypes serve as valuable tools for validating genome assemblies by analyzing conserved gene orders and chromosomal collinearity. They also facilitate the distinction between autopolyploids and allopolyploids, as evidenced in studies of *Cardamine chenopodiifolia* and *Saccharum officinarum* [5,6].

With the ongoing advancement of methods for reconstructing ancestral karyotypes, several ancestral karyotypes in angiosperms have been successfully established, including those for flowering plants, eudicot (AEK), Malvaceae (AMK), core Brassicaceae (ACBK), Asteraceae (AAK), bamboo (ABK), and grass (AGK) [2,7,8,9,10,11,12]. The construction of these karyotypes has significantly enhanced our understanding of the evolutionary history of karyotypes in angiosperms. However, the mass extinction event of gymnosperms—the other primary branch of seed plants—poses a challenge in identifying their most recent common ancestor [13,14]. As a result, reconstructing ancestral genomes through comparative analysis of extant gymnosperm genomes becomes crucial for elucidating the characteristics of early gymnosperm ancestors and their subsequent evolutionary trajectories.

Gymnosperms encompass four taxa, 13 families, and more than 1200 species that are distributed across both the northern and southern hemispheres [15]. This group includes economically essential species utilized in medicine and timber production, such as *Ephedra sinica*, *Taxus yunnanensis*, *Torreya grandis*, *Pinus massoniana*, and *Cunninghamia lanceolata*. Throughout the history of Earth, gymnosperms underwent a period of rapid speciation and predominantly thrived during the Mesozoic Era [16]. However, approximately 140 to 250 million years ago, the most recent common ancestor of all extant angiosperms experienced a rapid and extensive radial evolution that ultimately led to their dominance over gymnosperms as the primary plant group [17]. Despite angiosperms being recognized as the most diverse, widely distributed, and adaptable plant group today, coniferous forests composed of gymnosperms remain among the largest existing forest ecosystems. These forests hold considerable ecological and economic value [18]. Consequently, gymnosperms have emerged as important subjects of research across various disciplines, including forest ecology, plant breeding, genetics, physiology, and developmental biology. Research has significantly enhanced our understanding of unique characteristics, such as adaptability, phylogeny, and biogeographic patterns within gymnosperms through analyzing DNA sequences and genomes.

Most previous comparative genomics studies on gymnosperms have primarily focused on analyzing genomic collinearity and phylogenetic relationships among various species [16,19,20]. The publication of the *Cycas panzhihuaensis* genome [21], along with the availability of high-quality chromosome-level genomes from representative lineages of gymnosperms such as *Ginkgo biloba* [22], *T. yunnanensis* [23], *T. wallichiana* [24], *T. chinensis* var. *mairei* [25], *T. grandis* [26], *Metasequoia glyptostroboides* [27], *Sequoiadendron giganteum* [28], *P. tabulaeformis* [29], *Gnetum montanum* [30], and *Welwitschia mirabilis* [30], has provided a comprehensive resource for investigating the genome and chromosome evolution of gymnosperms as well as for reconstructing ancestral karyotypes of gymnosperms [31].

In this study, we reconstructed the ancestral karyotype of gymnosperms through a comparative genomic analysis of five phylogenetically divergent species. Subsequent analyses of synteny and transposable elements (TEs) provided novel insights into the evolutionary mechanisms underlying specific syntenic blocks. Our findings provide a framework for future investigations into patterns of genetic diversity and genomic evolution in gymnosperms.

## 2. Results

### 2.1. Reconstruction of Ancestral Gymnosperm Karyotype

The genomic information of ten gymnosperm species across four taxa was collected to construct the ancestral karyotypes of gymnosperms (Table 1 and Table S1). The chromosome numbers for eight of these gymnosperm species are either 11 or 12, which are different from those observed in gnetophyte species. Synteny blocks within each species were further identified. In the *T. grandis* genome, we identified a total of 9207 collinear gene pairs distributed across 174 synteny blocks. In contrast, *T. yunnanensis* exhibited the smallest number of collinear genes within its genome, with only 2307 collinear gene pairs distributed across 92 synteny blocks (Table S2). Additionally, we identified intergenomic collinear genes between pairs of gymnosperm species. Upon comparing collinearity, we found that the *G. biloba* genome exhibited a greater abundance of intergenomic collinear genes than other species, with a count ranging from 854 to 9470 distributed across 132 to 547 synteny blocks (Table S3). Based on the number of genes present in synteny blocks between each gymnosperm species and *G. biloba*, five representative species were selected for further chromosome-level synteny analyses (Table 1).

**Figure 1:**
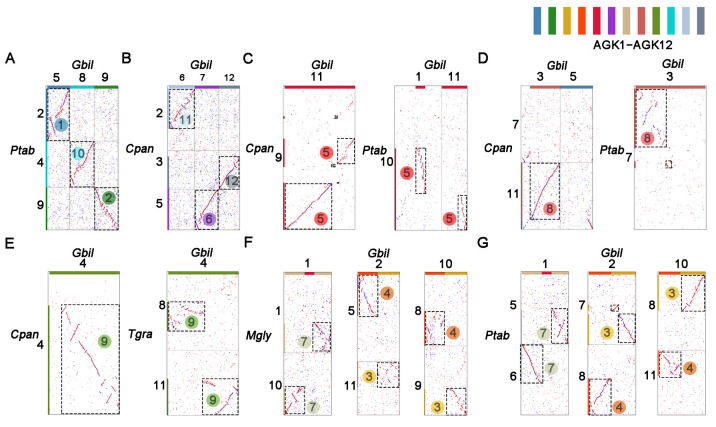
Inference of the ancestral chromosomes in gymnosperms. Collinearity blocks between pairs of gymnosperm species were identified to construct proto-chromosomes of AGK1 to AGK12, such as (**A**) AGK1, AGK2, and AGK10 from *G. biloba* (Gbil) vs. *P. tabulaeformis* (Ptab). (**B**) AGK11, AGK6, and AGK12 from *G. biloba* (Gbil) vs. *C. panzhihuaensis* (Cpan). (**C**) AGK5 from *G. biloba* (Gbil) vs. *C. panzhihuaensis* (Cpan) and *P. tabulaeformis* (Ptab). (**D**) AGK8 from *G. biloba* (Gbil) vs. *C. panzhihuaensis* (Cpan) and *P. tabulaeformis* (Ptab). (**E**) AGK9 from *G. biloba* (Gbil) vs. *C. panzhihuaensis* (Cpan) and *T. grandis* (Tgra). AGK3, AGK4, and AGK7 from *G. biloba* (Gbil) vs. *M. glyptostroboides* (Mgly) (**F**) and *P. tabulaeformis* (Ptab) (**G**). AGK1 to AGK12: the inferred gymnosperm proto-chromosomes were color-coded with 12 distinct colors to differentiate the chromosomes (SteelBlue, ForestGreen, GoldEnrod, OrangeRed, Crimson, DarkOrchid, Tan, IndianRed, OliveDrab, DarkTurquoise, LightSteelBlue, and SlateGray). Numbers with circular symbols represented ancestral chromosomes, and boxes of dotted lines were used to represent blocks involved in the construction of ancestral chromosomes.

All ancestral chromosomes are designated based on the number of genes they encompass, with AGK1 having the highest gene count. In the initial phase, the ancestral chromosomes were constructed from complete chromosomes that demonstrate synteny between the two species. As shown in Figure 1A, the Gbil5, Gbil8, and Gbil9 chromosomes of *G. biloba* are homologous to the Ptab2, Ptab4, and Ptab9 chromosomes of *P. tabuliformis*, respectively. Excluding inversions that do not affect the inference of the ancestral chromosome, these chromosome pairs exhibit homology across their entire lengths. Consequently, they may be derivatives of an ancestral chromosome and retain primary structures denoted as AGK1, AGK2, and AGK10 (Figure 1A). Another example highlights the inference of ancestral chromosomes AGK11, AGK6, and AGK12, where Gbil7 is homologous to Cpan5 across the entire chromosome. Meanwhile, Gbil6 and Gbil12 display homology with most regions of chromosomes Cpan2 and Cpan3, respectively. Although ginkgo and cycad are sister taxa in phylogenetic analyses, considering their early divergence as gymnosperms, we propose that Gbil6, Gbil12, and Gbil7 (Cpan5) are derived from three distinct ancestral chromosomes, referred to as AGK6, AGK11, and AGK12, respectively (Figure 1B).

In our analysis of the ancestral chromosome AGK5, we observed that the entirety of Gbil11 is homologous to a significant portion of Cpan9. However, it is essential to note that Gbil11 does not represent a complete ancestral chromosome. In the homologous gene dot plot between *P. tabuliformis* and *G. biloba,* we found that the second half of Ptab10 exhibits homology with a small section of Gbil1 as well as part of Gbil11. Furthermore, this arm in Ptab10 is conserved in *T. grandis* and *M. glyptostroboides* (Figure S1). Therefore, we inferred that these segments may represent derivatives from the ancient chromosome designated as AGK5 (Figure 1C).

In the second round of analysis, ancestral chromosome karyotypes were constructed from syntenic blocks of sub-chromosomes. During the analysis, we focused on syntenic blocks exhibiting consistent collinearity relationships with at least three species. A portion of chromosome Cpan11 in *C. panzhihuaensis* and a segment of chromosome Ptab7 in *P. tabuliformis* exhibited collinearity with chromosome Gbil3 in *G. biloba*. Consequently, we inferred that Gbil3 is derived from an ancestral chromosome designated as AGK8 (Figure 1D). Similarly, Gbil4 is likely derived from another ancestral chromosome labeled AGK9 (Figure 1E). The remaining three chromosomes, including Gbil2, Gbil10, and Gbil1, exhibit arm-level collinearity within the genomes of *M. glyptostroboides* (Figure 1F) and *P. tabuliformis* (Figure 1G). Based on the collinearity with *C. panzhihuaensis*, these three chromosomes were denoted as AGK3, AGK4, and AGK7, which is further supported by collinearity analysis with PGK (Figures S1 and S2A).

Finally, chromosome-level syntenic blocks were integrated to construct an ancestral gymnosperm karyotype (AGK) consisting of 12 proto-chromosomes. Most of the ancestral chromosomes were derived from *G. biloba*. In addition, the majority of AGK1 to AGK5 originated from *P. tabuliformis*, and AGK6 was sourced from *C. panzhihuaensis*. Collectively, the 12 ancestral chromosomes encompassed a total of 42,580 genes, including 18,058 genes from *G. biloba*, 21,891 genes from *P. tabuliformis*, and 2631 genes from *C. panzhihuaensis*, which provided a comprehensive representation of the genetic information within the analyzed species.

### 2.2. Karyotype Mapping and Collinearity Analyses of Gymnosperms

**Figure 2:**
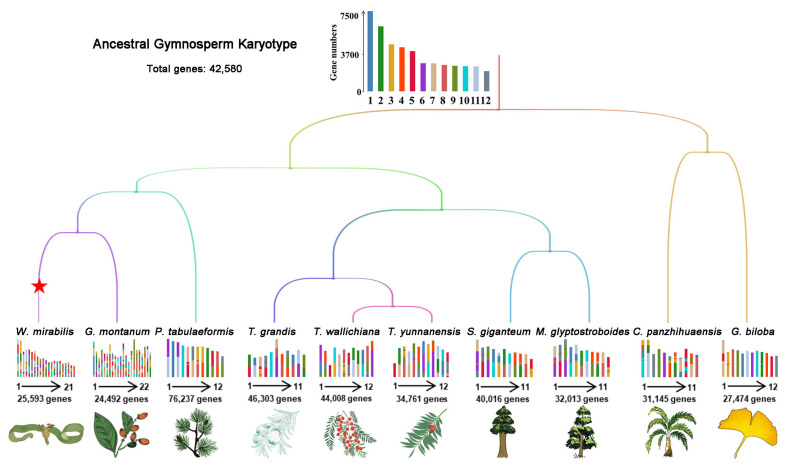
Karyotype projection of 10 extant gymnosperms based on AGK. The colors of the 12 proto-chromosomes are the same as in Figure 1. Whole-genome duplication events are represented by a red pentagram. The karyotypes of ancestral chromosomes in modern plant genomes are shown at the branches. The numbers of genes and chromosomes are exhibited for each species.

We further conducted karyotype mapping of AGK to the chromosomes of extant plants from four gymnosperm taxa (Figure 2). Cycads and ginkgo, recognized as the earliest diverging lineages among gymnosperms, hold significant importance in elucidating the evolution of the gymnosperm genome. Our study revealed that *G. biloba* retains ancestral karyotypic features across most chromosomes, whereas *C. panzhihuaensi* exhibits considerable chromosomal rearrangements, with only three conserved chromosomes preserving ancestral characteristics.

In conifers, we observed a significant conservation of karyotypes among various genera within the family, particularly between *Metasequoia* and *Sequoiadendron*. Gnetophytes, including *G. montanum* and *W. mirabilis*, have undergone considerable chromosomal rearrangements following their divergence from other lineages of gymnosperms (Figure 2).

**Figure 3:**
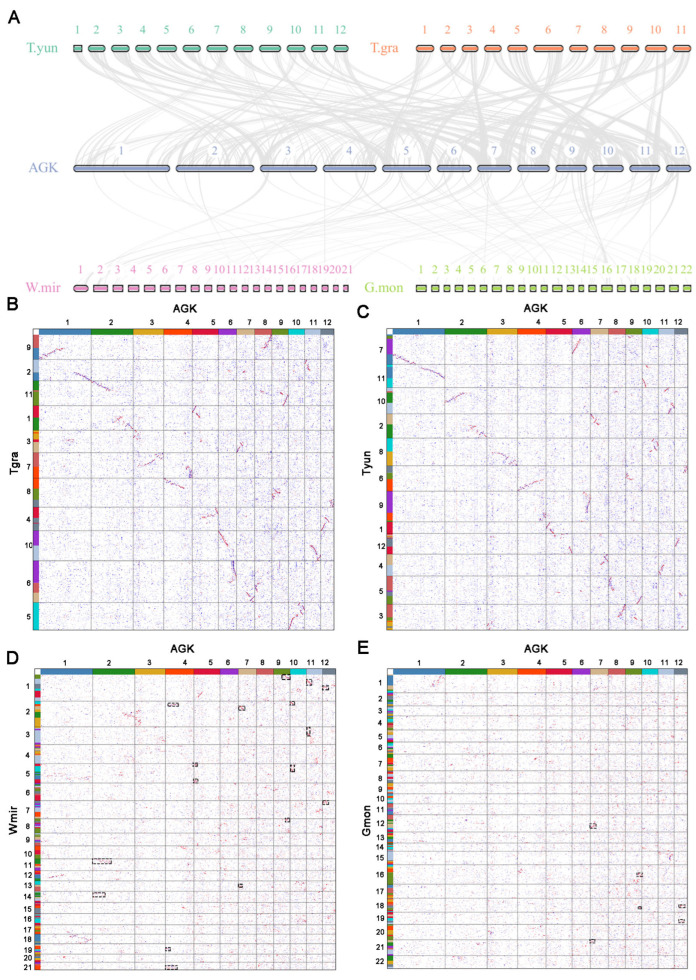
Synteny analysis of AGK and genomes of gymnosperm species. (**A**): Syntenic plot illustrating the relationship between AGK and various gymnosperms with differing chromosome numbers. The gray lines show collinear blocks. (**B**–**E**): Karyotype projection of four species genomes based on AGK, including *T. grandis* (**B**), *T. yunnanensis* (**C**), *W. mirabilis* (**D**), and *G. montanum* (**E**). Boxes with black dashed lines represent syntenic blocks that may have resulted from whole-genome duplication events. The colors of the 12 proto-chromosomes are the same as in Figure 1.

The reconstructed AGK was utilized for collinearity analysis with extant gymnosperms, revealing notable variations in the degree of collinearity (Figure 3 and Figure S3, Table S4). A strong collinearity was identified between AGK and species with chromosome numbers 11 and 12, indicating that these species have retained a higher degree of gene order conservation and evolutionary stability (Figure 3A–C). In contrast, gnetophyte plants with chromosome numbers of 21 or 22 (e.g., Wmir and Gmon) exhibited reduced levels of collinearity (Wmir: 6.3 gene pairs per block; Gmon: 6.5 gene pairs per block) (Figure 3A,D,E). The chromosome number in gnetophyte plants is approximately double that found in other gymnosperms, suggesting the possibility of a whole-genome duplication (WGD) event. Remarkably, despite these multiple chromosomal rearrangements (Figure 2), *W. mirabilis* exhibits partial karyotypic conservation, with Wmir18 containing conserved segments that are homologous to AGK (Figure 3D). The karyotype of *W. mirabilis* also displayed a 2:1 syntenic depth ratio when compared with AGK and *G. biloba* (Figure 3D and Figure S1). Furthermore, the *W. mirabilis* genome exhibited a 1:1 syntenic depth ratio within its own genomic structure, with a peak Ks for paralogous homologous genes estimated at approximately 1.12 (Figures S4A and S5). These findings suggest that *W. mirabilis* has indeed undergone a WGD event. In contrast, no distinct pattern of collinearity was observed in the *G. montanum* genome (Figure 3E and Figure S4B). Nevertheless, specific patterns of collinearity between *G. montanum* and both AGK and *G. biloba* support the hypothesis that *G. montanum* may have also experienced a WGD event (Figure 3E and Figure S1) [31].

To determine whether the decrease in collinearity observed in Gnetophytes is clade-specific, we compared the genome of autohexaploid *Sequoia sempervirens* with the constructed AGK. The results indicated that the degree of collinearity between *S. sempervirens* and AGK (Ssem: 7.4 gene pairs per block) was comparable to that between gnetophytes and AGK, and both were significantly lower than those observed in other gymnosperms with chromosome numbers of either 11 or 12 based on statistics from ANOVA (Figure S6, Table S4). Consequently, we propose that the chromosome structures of gymnosperm species are relatively stable, except in the species with multiplied chromosome numbers.

### 2.3. Distribution of Duplicated Blocks in Species with Multiplied Chromosome Numbers

To investigate the retention patterns of genes during chromosome rearrangement, duplicated genomic blocks were identified for gymnosperm species with multiplied chromosome numbers. A total of 177, 24, and 29 synteny blocks were obtained for *S. sempervirens*, *W. mirabilis*, and *G. montanum*, respectively. These synteny blocks were subsequently mapped onto the 24 chromosomal arms of the AGK (Figure S2B).

**Figure 4:**
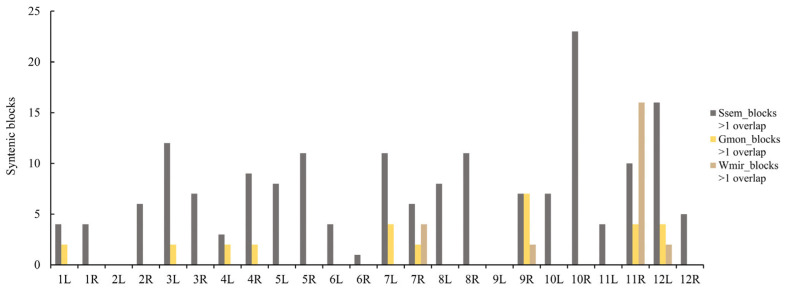
Distribution statistics of evolutionarily conserved syntenic blocks in the genomes of *S. sempervirens* (Ssem), *G. montanum* (Gmon), and *W. mirabilis* (Wmir) across the arms of the AGK chromosome.

Our analysis revealed variations in the number of syntenic blocks across these chromosomal arms. Syntenic blocks in *S. sempervirens* were identified on 22 chromosomal arms of AGK, with AGK10R containing 23 conserved syntenic blocks. In contrast, syntenic blocks in *W. mirabilis* were found on only four chromosomal arms of AGK, with AGK11R harboring 16 conserved syntenic blocks (Figure 4). In *G. montanum*, the distribution pattern of syntenic blocks exhibited a dispersed arrangement. Seven blocks demonstrated syntenic relationships with chromosomal arm AGK9R. Chromosomal arms AGK7L, AGK11R, and AGK12L each contained four collinear blocks (Figure 4). Our results indicate that, despite extensive chromosomal rearrangements during genome evolution, the conserved syntenic blocks exhibited non-random distributions across the AGK chromosomes.

To identify genomic features potentially associated with syntenic conservation, we conducted repeat sequence identification in both filtered syntenic blocks and their adjacent genomic regions (30-gene windows). Comparative analysis between syntenic blocks and their adjacent non-syntenic regions revealed distinct repeat composition patterns in *W. mirabilis* syntenic blocks, characterized by enrichment of Copia and LINE1 elements with concomitant depletion of Gypsy elements. In contrast, no distinguishable differences in transposable element composition were detected between syntenic blocks and their adjacent regions in *G. montanum*, with all TE categories being quantitatively comparable in distribution patterns (Table S5).

### 2.4. Enrichment of LINE1 Elements in Retained Genomic Blocks in the W. mirabilis Genome

**Figure 5:**
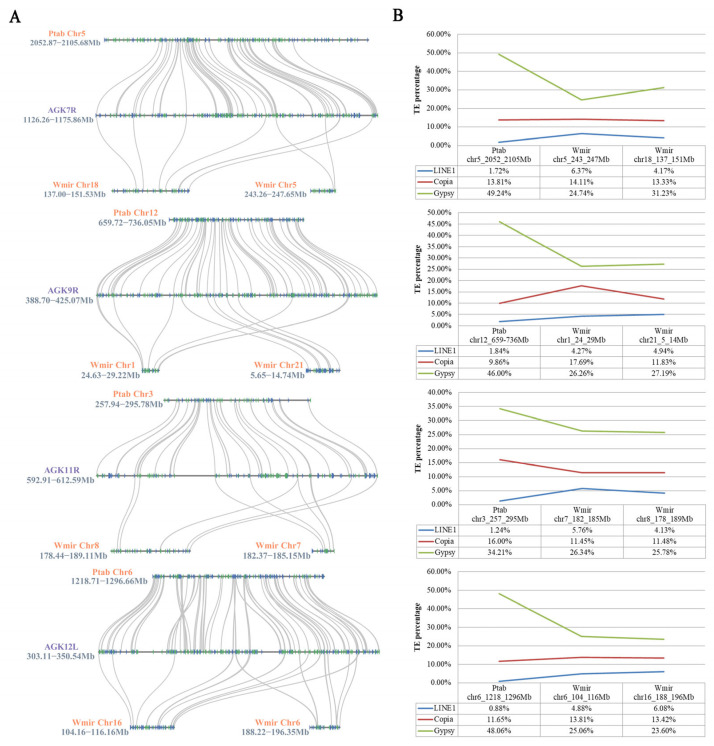
Comparative analysis of *W. mirabilis* and *P. tabuliformis* within the conserved syntenic block on AGK7R, AGK9R, AGK11R, and AGK12L. (**A**) Collinearity analysis of *W. mirabilis* and *P. tabuliformis* in the conserved syntenic block on AGK7R, AGK9R, AGK11R, and AGK12L. Orange font represents the chromosomes of the species, purple font represents the chromosome arms of AGK, and black font represents the position of the block on the chromosome. (**B**) Transposable element statistics of *W. mirabilis* and *P. tabuliformis* in the conserved syntenic block on AGK7R, AGK9R, AGK11R, and AGK12L.

To investigate the relationship between syntenic blocks and transposon content in the *W. mirabilis* genome, we selected *P. tabuliformis*, the phylogenetically closest extant relative to *W. mirabilis*, as a control. Comparative genomic analysis revealed that the syntenic blocks in *W. mirabilis* exhibit a more dispersed collinearity pattern with AGK at the gene level when compared with *P. tabuliformis* (Figure 5A). Analysis of transposon composition within these syntenic blocks identified Copia, Gypsy, and LINE1 as the three predominant transposon types. While the Copia elements showed comparable abundance between the syntenic regions of *P. tabuliformis* and *W. mirabilis*, it was observed that *P. tabuliformis* displayed a two-fold greater accumulation of Gypsy elements relative to *W. mirabilis*. Distinct distribution patterns for LINE1 emerged between these syntenic blocks, with *W. mirabilis* exhibiting marked LINE1 accumulation (4.13~6.37%), contrasting with the minimal representation in *P. tabuliformis* (0.88~1.84%) (Figure 5B). Such divergent distribution patterns for LINE1 correspond with *W. mirabilis*’s unique genomic architecture, particularly its enhanced propensity for complex gene repositioning events and macrosyntenic reshuffling.

### 2.5. Characteristics of Blocks in Angiosperms That Are Collinear with AGK

**Figure 6:**
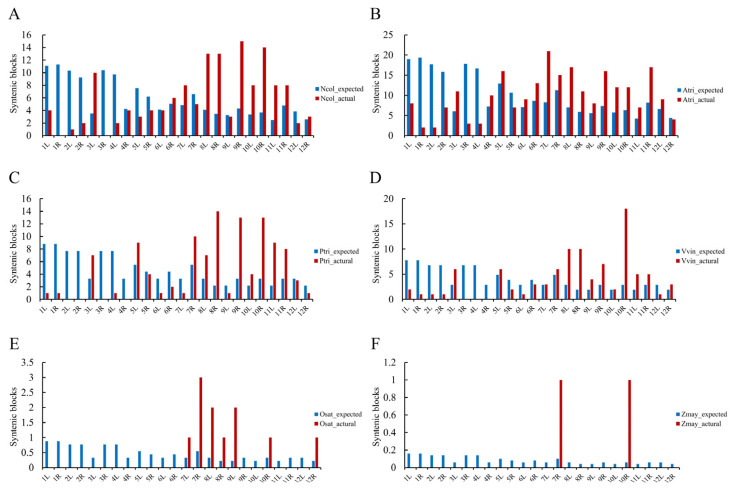
Distribution statistics of the synteny blocks on the arms of the AGK chromosome in *A. trichopoda* (**A**), *N. colorata* (**B**), *P. trichocarpa* (**C**), *V. vinifera* (**D**), *O. sativa* (**E**), and *Z. mays* (**F**), respectively. Blue bars represent the expected numbers of synteny blocks calculated according to the lengths of chromosomes, and red bars represent the actual number of synteny blocks presented on the chromosome arms.

The constructed AGK was subsequently employed for collinearity analysis with the ancestral angiosperm karyotype and angiosperm species, revealing the conservation of genomic regions despite the divergence that has occurred over millions of years (Figure S7). In our investigation, two basal angiosperms, two core dicotyledons, and two monocots were selected. The identification of the syntenic blocks indicated that *A. trichopoda* and *N. colorata* possess 238 and 141 conserved blocks shared with AGK, respectively, which are distributed across all 24 chromosome arms of AGK. In contrast, divergent species displayed substantially reduced conservation, with 110 in *Populus trichocarpa*, 97 in *Vitis vinifera*, 11 in *Oryza sativa*, and 2 in *Zea mays*, and their distribution was restricted to subsets of AGK chromosome arms. The reduced number of conserved blocks during species divergence further suggests that AGK serves as an appropriate framework for comparative genomic analyses across basal angiosperm lineages. Furthermore, a randomization model was developed based on the proportions of chromosomal arm lengths to simulate syntenic block allocation and assess the randomness of their distribution. No significant association between chromosomal length and syntenic block positioning in six angiosperms (Figure 6), suggesting a non-random positional bias in the arrangement of these conserved blocks within AGK.

## 3. Discussion

### 3.1. Ancestral Chromosomes of Gymnosperms

The construction of AGK is pivotal for understanding the evolution of gymnosperms. The reconstruction of the AGK using paleogenomic approaches has become feasible because of the availability of high-quality diploid genomes representing nearly all major clades. Due to extensive chromosomal rearrangements within gnetophytes, this clade was excluded from AGK reconstruction. The five representative species selected phylogenetically cover all three gymnosperm taxa: cycads, ginkgo, and conifers. Given the karyotypic stability observed in most existing gymnosperms, utilizing these five species to construct the ancestral karyotype is reliable. We inferred the ancestral karyotype of gymnosperms at shared evolutionary nodes, which aligns with the previously published proto-gymnosperm karyotype (PGK) [31]. Future efforts should expand taxonomic sampling to include more early-diverging gymnosperm lineages, such as additional cycads, gnetophytes, *Araucariaceae*, or *Podocarpaceae*, which will help enhance the robustness of AGK reconstruction. Meanwhile, telomere-to-telomere (T2T) genome assemblies will improve AGK accuracy and deepen our understanding of centromere and chromosome structure.

Our analysis demonstrated the significant preservation of ancestral karyotype features and chromosome number in *G. biloba*, indicating that the AGK remained evolutionarily stable for a considerable period following the origin of gymnosperms, making it a valuable resource for understanding the evolution and biology of early gymnosperms. Comparative analyses revealed multiple rearrangements in *C. panzhihuaensis* relative to the AGK, supporting its status as a descendant of ancient lineages, aligning with previous research [32,33,34,35]. However, a comprehensive analysis of shared chromosomal events and evolutionary relationships is appropriate for more detailed ancient chromosomes, as demonstrated in the *Brassicaceae* study by Jiang et al. [36]. Furthermore, despite millions of years of divergence between gymnosperms and angiosperms, non-randomly distributed synteny blocks can still be observed in pairwise comparisons, which is consistent with the results observed by previous collinearity analyses between the *G. biloba* genome and those of *A. trichopoda* and *N. colorata* [37]. Notably, functional homologous gene copies in conifers are preferentially enriched on the same chromosomes, contrasting with the dispersed distribution patterns characteristic of angiosperms [38]. Therefore, we speculate that the non-random positional bias in this conserved block is related to functional conservation. Future studies should expand taxonomic sampling across diverse angiosperm lineages to test this hypothesis.

### 3.2. Chromosome Stability and Chromosome Numbers

Our study revealed a significant decrease in chromosome collinearity with AGK in species with multiplied chromosome numbers. Various mechanisms may contribute to the observed phenomenon. Genome evolution is a continuous and dynamic process that occurs over extended periods of time. Following polyploidy events that occur in species such as *S. sempervirens* and *W. mirabilis*, the genomes may undergo rediploidization, resulting in the widespread loss of genes and/or dramatic recombination of chromosomes. As a result, their collinearity with homologous chromosomes in other species diminishes gradually over time [39,40,41,42]. Furthermore, despite the absence of recent whole-genome duplication events in *G. montanum* [30,43], its chromosome numbers closely resemble those of *W. mirabilis.* Given the rapid evolutionary rate of *G. montanum* [44], we speculate that it may have masked WGD events, as reported in the *Ceratopteris richardii* genome [45]. In contrast, *W. mirabilis* has undergone a recent whole-genome duplication event [30], which may have resulted in incomplete rediploidization because of a slower diploidization process. This hypothesis is supported by genomic studies on hexaploid *S. sempervirens* [46]. These processes could lead to drastic rearrangements or even the loss of chromosomes within these species. Future research should aim to expand genomic data on gnetophytes and explore whether *G. montanum* experienced descending dysploidy followed by rediploidization, ultimately leading to a reduction in chromosome number.

Genome downsizing is a crucial mechanism to counteract genome expansion resulting from polyploidy or repeat amplification [4,40,47]. Gymnosperms generally exhibit large genomes with a significant proportion of transposons. However, gnetophyte plants have relatively small genome sizes, which may be attributed to the removal of transposons. Increased gene loss and translocation could occur during the process of transposon removal. Desert plants such as *W. mirabilis*, which inhabit extremely harsh environments, may render their genomes more susceptible to the effects of environmental stress. For example, exposure to intense ultraviolet radiation may have influenced chromosome dynamics, resulting in frequent rearrangements [30]. The harsh environments could impact meiosis processes, such as synapsis and segregation of chromosomes. It has been reported that both elevated and reduced temperatures increase the frequency of crossover events and regulate chromosome organization [48]. In short, a strong correlation between chromosome stability and chromosome number exists in gymnosperms; however, the underlying molecular mechanisms have not yet been fully elucidated. Previous studies have examined the high frequency of polyploidy in the genera *Ephedra* and *Juniperus* within gymnosperms [49,50]. The availability of additional high-quality polyploid genomes in gymnosperms will enhance our understanding of the relationship between chromosome stability and chromosome number.

Methodological advancements with emerging synteny graph techniques are also crucial for the reconstruction of ancestral karyotypes. GENESPACE serves as an analytical pipeline for delineating synteny and orthology patterns across multiple genomes [51]. Oxford Dot Plots are specifically designed to compare chromosomal evolution among species by leveraging chromosome collinearity data, thereby facilitating the inference of evolutionary relationships and identifying ancestral syntenic groups based on chromosomal-scale genome assemblies [52]. Syntenet reconstructs synteny networks from genome-wide protein sequences, revealing both highly conserved synteny clusters and taxa-specific clusters [53]. The integration of these complementary methods will expedite our understanding of biological evolution and genome structure by employing distinct computational paradigms for synteny analysis.

### 3.3. Potential Roles of Repetitive Sequences in Maintaining Chromosome Stability

The proportion of repetitive sequences in gymnosperms is generally higher than that observed in angiosperms, which aligns with the larger genome sizes characteristic of gymnosperms [54]. The centromeric and telomeric regions of chromosomes are predominantly composed of highly repetitive structural sequences. Our findings indicate that the chromosome structure of most gymnosperms exhibits relatively high stability, contrasting with that observed in angiosperms. As a key regulator of chromosome segregation, centromeric DNA is regarded as a “Trojan horse” maintaining genome stability [55]. Studies on conifers have revealed that centromeric–centromeric reciprocal translocations serve as the predominant pattern of chromosome evolution, while telomeric–centromeric reciprocal translocations represent one of the primary mechanisms driving chromosomal reduction in Cupressales [31]. During chromosome evolution, the number of chromosomes in gymnosperms is relatively stable, suggesting the number of primary centromeres is well maintained in gymnosperms during chromosome rearrangement [56].

Telomeres and retrotransposons, both of which are repetitive elements, exhibit mutual regulatory interactions that safeguard chromosomal integrity. Activation of Ty1 LTR retrotransposons has been demonstrated to promote telomere stabilization in yeast [57]. Conversely, retrotransposons such as LINE1, which are activated following telomere shortening, may facilitate the partial restoration of telomere length by indirectly activating telomerase expression [58]. Furthermore, insertional mutagenesis by LINE1 has been documented to induce complex chromosomal translocations and large-scale genomic rearrangements [59]. Particularly, LINE1 elements can modulate gene expression through various mechanisms, including functioning as promoters or enhancers, disrupting transcript splicing, and altering epigenetic modifications, which may potentially compromise genomic stability [60,61,62]. In our study, we observed a higher proportion of LINE1 elements within syntenic blocks in *W. mirabilis*. This finding may contribute to the weakened synteny observed during chromosome rearrangement. Given that telomeric regions remain undetected in *W. mirabilis* [30], future studies utilizing higher-quality genomic data will be essential for evaluating the effects of LINE1 on chromosome stability and examining potential associations between LINE1 abundance and telomere dynamics.

In conclusion, we inferred that the AGK comprises 12 chromosomes in this study. Future advancements in reconstructing the ancestral gymnosperm genome and elucidating the evolutionary trajectory of gymnosperms will be enhanced by incorporating additional genomes from early-diverging lineages. By including a broader array of gymnosperm genomes in our research, a more comprehensive understanding of their evolution will be achieved. With ongoing progress in assembly technologies and decreasing sequencing costs, the accurate assembly of repetitive regions, such as centromeric and telomeric regions of chromosomes, will provide further insights into the relationship between repetitive sequences and chromosome stability within gymnosperms.

## 4. Materials and Methods

### 4.1. Data Sources

The chromosome-level genome assemblies, protein sequences, and genome annotation information were downloaded from public databases, including the China National GeneBank DataBase (https://db.cngb.org/; accessed on 5 June 2024), the China National Center for Bioinformation (https://www.cncb.ac.cn/; accessed on 5 June 2024), and the TreeGenes (https://treegenesdb.org/; accessed on 5 June 2024). The accessions and links to these data are listed in Table S1.

### 4.2. Reconstruction of AGK and Karyotype Projections

The reconstruction process of ancestral karyotypes involves two rounds, following the methodology described in previous studies [63]. Five representative species, including *G. biloba*, *C. panzhihuaensis*, *M*. *glyptostroboides*, *T*. *grandis*, and *P. tabuliformis*, were primarily utilized to reconstruct the ancestral karyotype of gymnosperms. Other gymnosperm species served as references for verification purposes. The analysis focused exclusively on genes located on chromosomes; genes on scaffolds were excluded from consideration. The construction of ancestral chromosomes was based on the collinearity of synteny blocks across these five representative species. Initially, similarity relationships among protein sequences of chromosomal genes were determined using BLASTP (v2.12.0) with an E-value threshold of 1 × 10^−5^ [64]. Subsequently, the WGDI toolkit was used to generate homologous gene dot plots with the parameter “-d” [63]. These dot plots were further analyzed to identify chromosomes that exhibited complete collinearity relationships between highly differentiated lineages.

The reconstruction of the AGK was conducted through a series of systematic steps. First, ancestral blocks at the chromosomal level were identified across all species. These homologous chromosomal segments were subsequently removed from each species. The remaining chromosomal segments were then reconnected and will continue to be utilized for identifying ancestral blocks until coverage of all chromosomes is achieved. When multiple species’ blocks are recognized as belonging to the same ancestral chromosome, we prioritize selecting a more continuous block from one of these species to serve as the representative for that ancestral block. Partial homologies were consolidated into a single ancestral chromosome if supported by genomes from at least two species and not contradicted by any other species. Once all ancestral chromosomes have been delineated, the chromosome composition of each extant species can be inferred based on pairwise relationships among species using WGDI with the parameter “-km”. By employing this methodology, we successfully obtained the final AGK. The chromosomal arms of AGK were determined through alignment with PGK centromeric loci [31].

### 4.3. Collinearity Analyses

We identified syntenic blocks between the two species using WGDI with the parameters “-icl” and “-bi”. The counts of gene pairs and homologous blocks were conducted following the removal of ancient collinear fragments using WGDI with the parameter “-c”. The ratio of gene pairs to syntenic blocks was used to assess the degree of collinearity between AGK and each species. MCscan (Python version, https://github.com/tanghaibao/jcvi/wiki/, accessed on 12 July 2024) [65] was used to analyze and visualize the genomic synteny of multiple species.

### 4.4. Repeat Annotation

To eliminate potential biases introduced by methodological variations in annotation protocols, all transposable elements within syntenic blocks examined in this study were rigorously identified and annotated using a unified set of standardized criteria. The accurate identification of LTR retrotransposons necessitated a multi-stage annotation protocol, beginning with complementary detection of candidate elements using LTRharvest v1.6.2 [66] with parameter “-similar 90, -vic 10, -seed 20, -seqids yes, -minlenltr 100, -maxlenltr 7000, -mintsd 4, -maxtsd 6, -motif TGCA, -motifmis 1” and LTR_FINDER v1.0.7 [67] with default parameters. Consensus integration of these predictions was subsequently performed using LTR_retriever v2.9.0 [68] with default parameters. A species-specific transposon library was constructed using the SINE/LINE database (https://sines.eimb.ru/) with manual correction and the EDTA v2.2.1 [69] pipeline (perl EDTA.pl -sensitive 1, -anno 1, -curatedlib). Unclassified LTR retrotransposons were subsequently categorized using DeepTE [70] with the parameter “python DeepTE.py -sp P -m_dir Plants_model -fam LTR”. The final non-redundant repeat library was established through hierarchical clustering and manual curation to eliminate sequence redundancy.

## Figures and Tables

**Table 1:** Statistics of genome assemblies and gene models for gymnosperm species.

Taxa	Species ^a^	Total Chromosomes	Genes on Chromosomes	Genome Size (Gb)
Cycads	*Cycas panzhihuaensis* (R)	*n* = 11	31,145	10.48
Ginkgophyte	*Ginkgo biloba* (R)	*n* = 12	27,474	9.88
Conifiers	*Metasequoia glyptostroboides* (R)	*n* = 11	32,013	8.07
	*Sequoiadendron giganteum*	*n* = 11	40,016	8.13
	*Torreya grandis* (R)	*n* = 11	46,303	19.05
	*Taxus yunnanensis*	*n* = 12	34,761	10.73
	*Taxus wallichiana*	*n* = 12	44,008	11.12
	*Pinus tabulaeformis* (R)	*n* = 12	76,237	25.42
Gnetophytes	*Gnetum montanum*	*n* = 22	24,492	4.14
	*Welwitschia mirabilis*	*n* = 21	25,593	6.87

^a^ “R” in parentheses indicates representative species selected for the reconstruction of ancestral chromosomes.

## Data Availability

The original contributions presented in this study are included in the article/Supplementary Material. Further inquiries can be directed to the corresponding author.

## Supplementary Materials

The following supporting information can be downloaded at: https://www.mdpi.com/article/10.3390/plants14152361/s1, Figure S1: Dot plots of homologous genes in ten gymnosperm species; Figure S2: Dot plots of homologous genes between ancestral gymnosperm karyotype (AGK) and proto-gymnosperm karyotype (PGK); Figure S3: AGK-based karyotype projection of six chromosome-level genomes including C. panzhihuaensis, G. biloba, P. tabulaeformis, S. giganteum, M. glyptostroboides, and T. grandis; Figure S4: Intra-genomic synteny for W. mirabilis (A) and G. montanum (B). Boxes of black-dashed lines represent syntenic blocks; Figure S5: Histogram showing the distribution of Ks of paralogous genes in W. mirabilis genome; Figure S6: Synteny analysis between AGK and S. sempervirens; Figure S7: Dot plots of homologous gene between AGK and ancestral angiosperm karyotype (AAK), Amborella trichopoda and Nymphaea colorata; Table S1: Data sources of the 17 species used in this study; Table S2: Numbers of colinear gene pairs and blocks within each of ten gymnosperm species; Table S3: Numbers of colinear gene pairs and blocks between G. biloba and nine gymnosperm species; Table S4: Summary of gene pairs in synteny blocks between AGK and eight gymnosperms, respectively; Table S5: Transposon composition in conserved syntenic blocks and adjacent 30 gene regions between AGK and W. mirabilis/G. montanum.
