# Supplementary material for: Construction of Ancestral Chromosomes in Gymnosperms and the Application in Comparative Genomic Analysis
Source: Plants (Basel). 2025 Aug 1;14(15):2361. doi: 10.3390/plants14152361 (PMC12349002; doi:10.3390/plants14152361)
Supplement: Supplementary file 1 [file plants-14-02361-s001.zip › plants-3772637-supplementary.pdf]

Supplementary Data

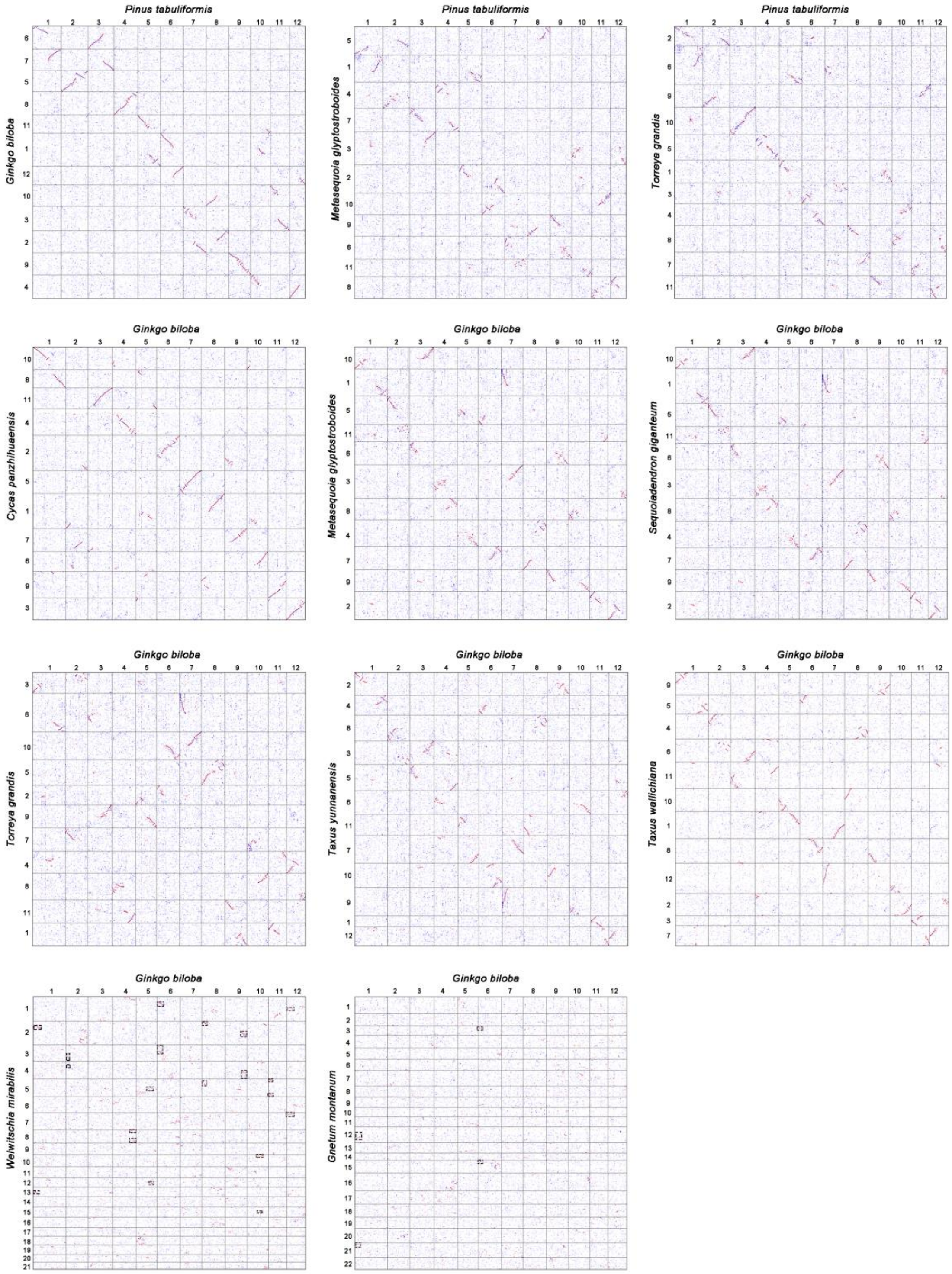

Figure S1: Dot plots of homologous genes in ten gymnosperm species. The genomes of *Ginkgo biloba*, *Metasequoia glyptostroboides,* and *Torreya grandis* were compared with the genome of *Pinus tabulaeformis.* The genomes of *Cycas panzhihuaensis*, *Metasequoia glyptostroboides, Sequoiadendron giganteum*, *Torreya grandis*, *Taxus yunnanensis*, *Taxus wallichiana*, *Welwitschia mirabilis* and *Gnetum montanum* were compare the genome of *Ginkgo biloba.*Boxes of black dashed lines represent syntenic blocks supporting whole genome duplication events.

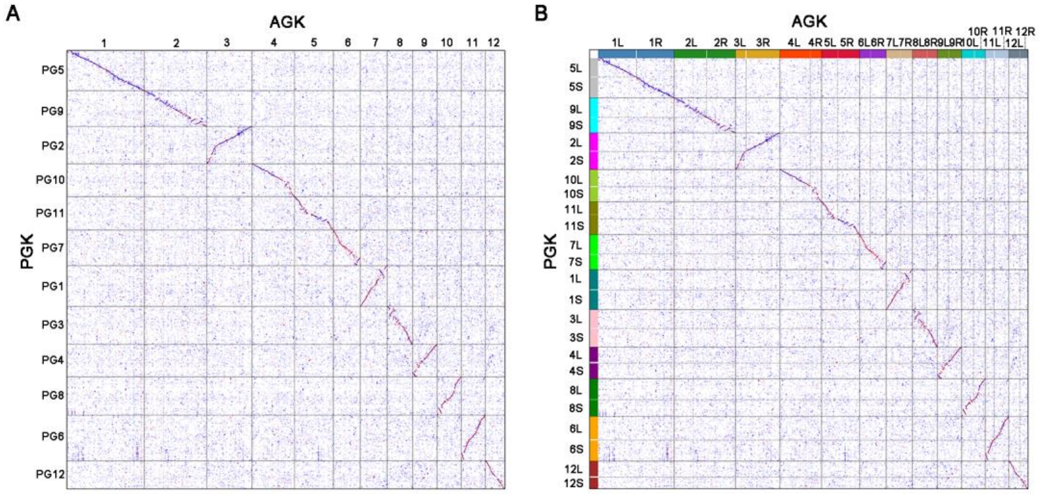

Figure S2: Dot plots of homologous genes between ancestral gymnosperm karyotype (AGK) and proto-gymnosperm karyotype (PGK). The results were presented at both chromosome level (A) and chromosome arm level (B). L and S in PGK represent to the long and short arms respectively; L and R in AGK denote the left and right arm accordingly.

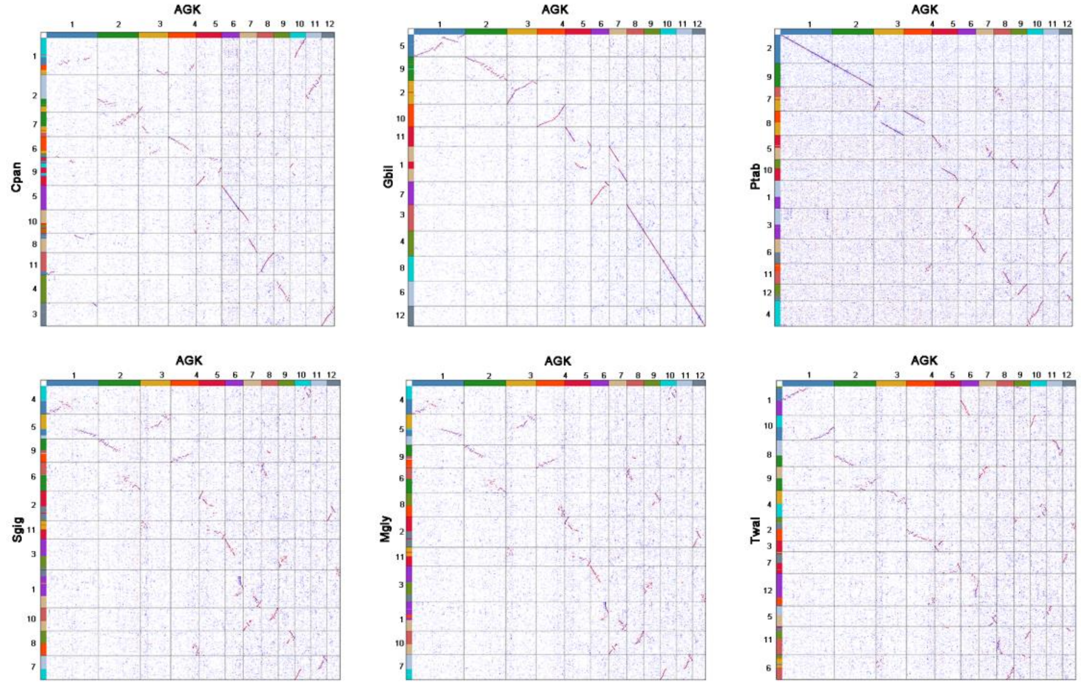

Figure S3: AGK-based karyotype projection of six chromosome-level genomes including *C. panzhihuaensis*, *G. biloba*, *P. tabulaeformis*, *S. giganteum*, *M. glyptostroboides*, and *T. grandis*.

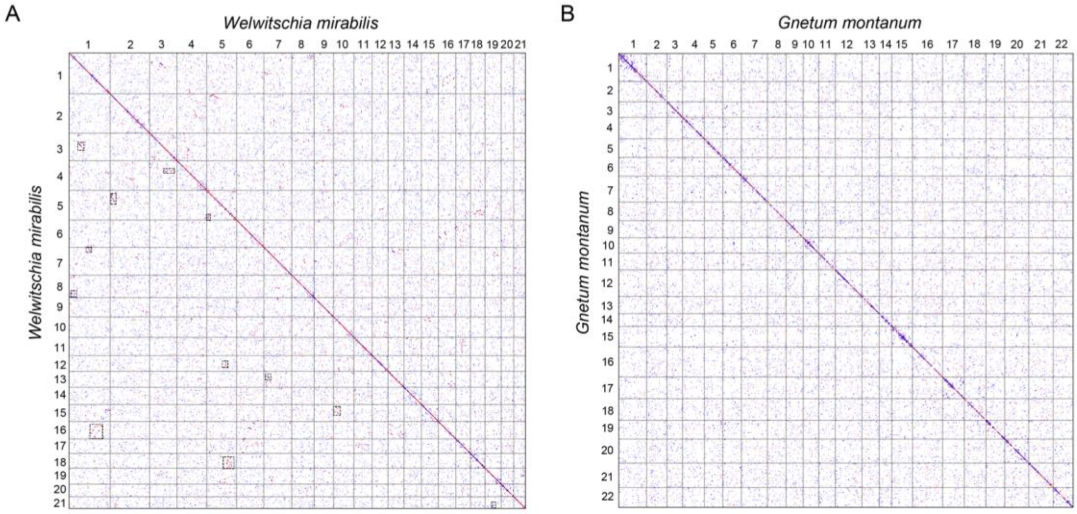

Figure S4: Intra-genomic synteny for *W. mirabilis* (A) and *G. montanum* (B). Boxes of black-dashed lines represent syntenic blocks.

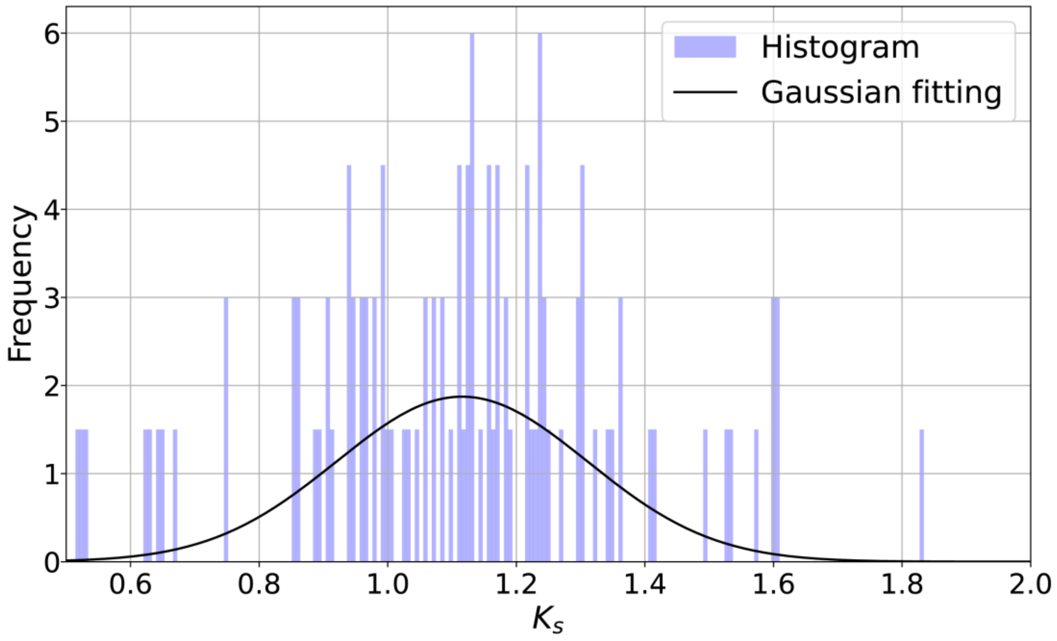

Figure S5: Histogram showing the distribution of *Ks* of paralogous genes in *W. mirabilis* genome.

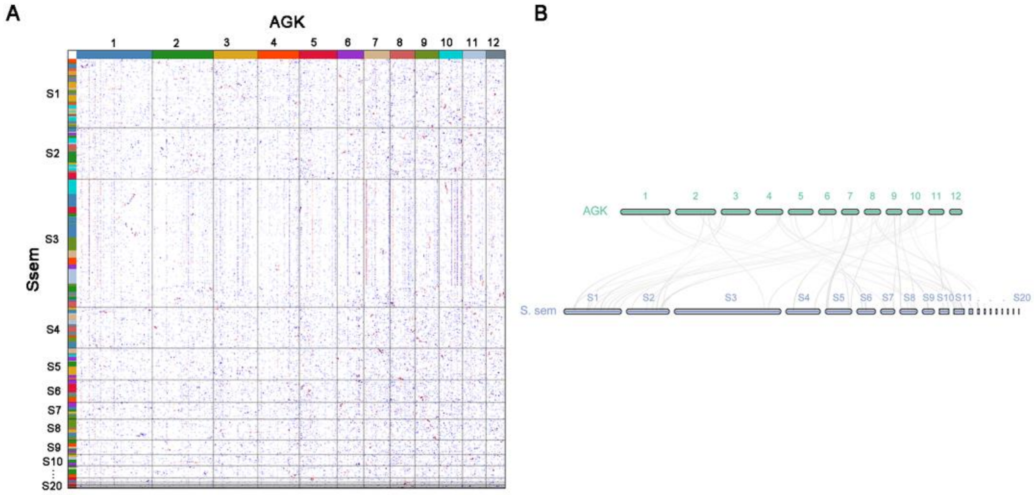

Figure S6: Synteny analysis between AGK and *Sequoia sempervirens*. Syntenic dot plot (A) and Synteny plot (B) between AGK and *S. sempervirens* (Ssem) were presented respectively. S in figure for *S. sempervirens* indicates scaffold.

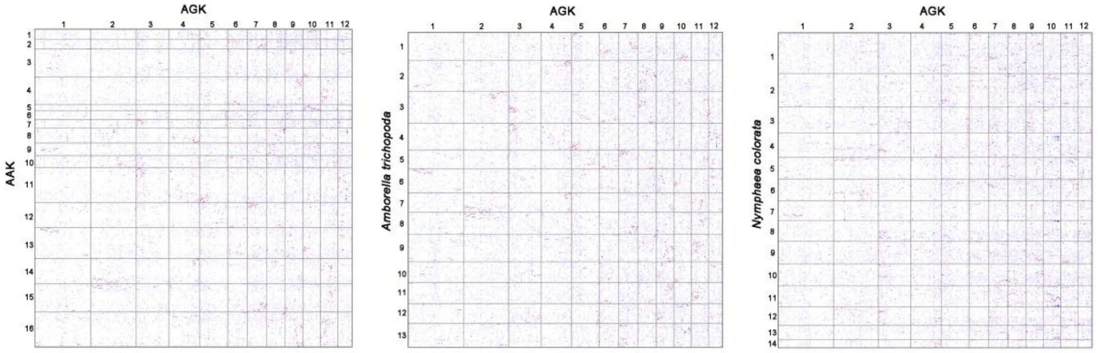

Figure S7: Dot plots of homologous gene between AGK and ancestral angiosperm karyotype (AAK), *Amborella trichopoda* and *Nymphaea colorata*.

Table S1: Data sources of the 17 species used in this study.

Species	Order	DOI	Data source
Cycas panzhihuaensis [1]	Cycadales	10.1038/s41477-022-01129-7	https://db.cngb.org/codeplot/datasets/public_dataset?id=PwRftGHfPs5qG3gE
Ginkgo biloba[2]	Ginkgoales	10.1038/s41477-021-00933-x	https://ngdc.cncb.ac.cn/bioproject/browse/PRJCA001755
Metasequoia glyptostroboides [3]	Cupressales	10.1016/j.xplc.2023.100643	https://ngdc.cncb.ac.cn/gwh/Assembly/37841/show
Sequoiadendron giganteum [4]	Cupressales	10.1534/g3.120.401612	https://treegenesdb.org/FTP/Genomes/Segi/v2.0/
Sequoia sempervirens [5]	Cupressales	10.1093/g3journal/jkab380	https://treegenesdb.org/FTP/Genomes/Sese/v2.1/
Taxus wallichiana [6]	Cupressales	10.1016/j.molp.2021.04.015	https://www.sciencedirect.com/science/article/pii/S167420522100160X?via%3Dihub
Taxus yunnanensis [7]	Cupressales	10.1038/s42003-021-02697-8	https://www.ncbi.nlm.nih.gov/bioproject/PRJNA661543/
Torreya grandis [8]	Cupressales	10.1038/s41467-023-37038-2	https://doi.org/10.6084/m9.figshare.21089869
Pinus tabulaeformis [9]	Pinales	10.1016/j.cell.2021.12.006	https://figshare.com/articles/dataset/Pinus_tabuliformis_gene_space_annotation/16847146/1
Gnetum montanum [10]	Gnetales	10.1038/s41467-021-24528-4	https://db.cngb.org/search/project/CNP0001943/
Welwitschia mirabilis [10]	Welwitschiales	10.1038/s41467-021-24528-4	https://db.cngb.org/search/project/CNP0001943/
Amborella trichopoda [11]	Amborellales	10.1038/s41477-024-01858-x	https://phytozome-next.jgi.doe.gov/
Nymphaea colorata [12]	Ranales	10.1038/s41586-019-1852-5	http://waterlily.eplant.org/
Populus trichocarpa [13]	Salicales	10.1126/science.1128691	https://phytozome-next.jgi.doe.gov/info/Ptrichocarpa_v4_1
Vitis vinifera[14]	Vitales	10.1038/nature06148	https://phytozome-next.jgi.doe.gov/info/Vvinifera_v2_1
Oryza sativa [15]	Poales	10.1093/nar/gkl976	https://phytozome-next.jgi.doe.gov/info/Osativa_v7_0
Zea mays [16]	Poales	10.1002/tpg2.20114	https://phytozome-next.jgi.doe.gov/info/ZmaysPHJ40_v1_2

Table S2: Numbers of colinear gene pairs and blocks within each of ten gymnosperm species.

Species	No. of Gene Pairs	No. of Blocks	Genes per block	Significant level
C. panzhihuaensis	4429	263	16.8	c
G. biloba	3918	292	13.4	c
M. glyptostroboides	5794	91	63.7	a
S. giganteum	7859	114	68.9	a
T. wallichiana	7018	135	52.0	a
T. yunnanensis	2307	92	25.1	c
T. grandis	9207	174	52.9	a
P. tabulaeformis	8205	230	35.7	b
G. montanum	2704	94	28.8	b
W. mirabilis	4945	137	36.1	b

Note: Different lowercase letters in the column of "Significant level" indicate significant difference ($p < 0.0001$).

Table S3: Numbers of colinear gene pairs and blocks between *G. biloba* and nine gymnosperm species.

Pairs of species	No. of Gene Pairs	No. of Blocks	Genes per block	Significant level
G. biloba vs *C. panzhihuaensis*	9470	254	37.3	a
G. biloba vs *M. glyptostroboides*	6708	422	15.9	b
G. biloba vs *S. giganteum*	6368	434	14.7	b
G. biloba vs *T. wallichiana*	6548	402	16.3	b
G. biloba vs *T. yunnanensis*	6100	400	15.3	b
G. biloba vs *T. grandis*	6896	450	15.3	b
G. biloba vs *P. tabulaeformis*	7905	547	14.5	b
G. biloba vs *G. montanum*	854	132	6.5	c
G. biloba vs *W. mirabilis*	1227	187	6.6	c

Note: Different lowercase letters in the column of "Significant level" indicate significant difference ($p < 0.0001$).

Table S4: Summary of gene pairs in synteny blocks between AGK and eight gymnosperms, respectively.

**Item**	**Mgly-AGK**	**Twal-AGK**	**Tyun-AGK**	**Tgra-AGK**	**Sgig-AGK**	**Ssem-AGK**	**Wmir-AGK**	**Gmon-AGK**
Gene pairs	6118	6457	5540	6137	5720	1800	1162	879
Block No.	443	459	421	449	439	243	185	137
Medium	5	5	5	5	5	5	5	5
Max	126	139	116	112	97	23	17	14
Genes per block	14.2	14.1	13.2	13.7	13.1	7.4	6.3	6.5
Significant level	a	a	a	a	a	b	b	b

Note: AGK: ancestral gymnosperm karyotype, Mgly: *Metasequoia glyptostroboides*, Twal*: Taxus wallichiana*, Tyun*: Taxus yunnanensis*, Tgra: *Torreya grandis,* Sgig*: Sequoiadendron giganteum*, Ssem*: Sequoia sempervirens*, Wmir*: Welwitschia mirabilis*, Gmon*: Gnetum montanum.* Different lowercase letters in the row of "Significant level" indicate significant difference ($p < 0.0001$).

Table S5: Transposon composition in conserved syntenic blocks and adjacent 30 gene regions between AGK and *W. mirabilis*/*G. montanum*.

Repeat Classes		***G. montanum***		***G. montanum* adjacent 30 gene regions**		***W. mirabilis***		***W. mirabilis* adjacent 30 gene regions**	
		**bpMasked**	**%masked**	**bpMasked**	**%masked**	**bpMasked**	**%masked**	**bpMasked**	**%masked**
Class I: Retrotransposon		100,017,098	62.96%	121,779,735	64.95%	111,390,565	43.02%	171,906,159	43.65%
LTR	Copia	4,103,359	2.58%	5,707,092	3.04%	33,162,429	12.81%	49,064,660	12.46%
	Gypsy	90,569,506	57.01%	109,290,401	58.29%	63,679,931	24.59%	106,823,123	27.12%
	Endogenous_Retrovirus	/	/	/	/	1,178,725	0.46%	304,404	0.08%
	unknown	517,183	0.33%	803,979	0.43%	547,963	0.21%	1,361,102	0.35%
LINE	L1	3,063,049	1.93%	3,616,355	1.93%	11,820,705	4.56%	12,562,340	3.19%
	L2	20,129	0.01%	/	/	/	/	/	/
	Other	1,743,872	1.10%	2,324,718	1.24%	867,819	0.34%	1574375	0.40%
SINE	tRNA	/	/	37,190	0.02%	132,993	0.05%	216,155	0.05%
Class II: DNA transposon		785,449	0.50%	640,919	0.34%	1,284,241	0.49%	2,678,818	0.68%
TIR	CACTA	272	0.00%	136	0.00%	346,676	0.13%	356,844	0.09%
	Mutator	216,429	0.14%	216,450	0.12%	/	/	133,561	0.03%
	POLE	/	/	/	/	82	0.00%	628	0.00%
	Tcl_Mariner	/	/	112	0.00%	/	/	/	/
	hAT	113,507	0.07%	138,792	0.07%	520,504	0.20%	1,244,256	0.32%
	polinton	/	/	/	/	84,598	0.03%	677,937	0.17%
	PIF_Harbinger	/	/	54165	0.03%	/	/	/	/
nonTIR	helitron	455,241	0.29%	231,264	0.12%	332,381	0.13%	265,592	0.07%
repeat_fragment		18,155,194	11.43%	18,497,410	9.87%	50,231,704	19.40%	77,133,958	19.58%
Total interspersed		119,161,945	75.00%	140,978,034	75.20%	163,056,329	62.97%	251,718,935	63.91%
Total Length		158,877,278		187,481,955		258,944,634		393,860,109	

References

1. Liu Y, Wang S, Li L, Yang T, Dong S, Wei T, Wu S, Liu Y, Gong Y, Feng X, et al. The Cycas genome and the early evolution of seed plants. *Nature Plants*, **2022**, 8 (4)**:** 389-401. https://doi.org/10.1038/s41477-022-01129-7.
2. Liu H, Wang X, Wang G, Cui P, Wu S, Ai C, Hu N, Li A, He B, Shao X, et al. The nearly complete genome of *Ginkgo biloba* illuminates gymnosperm evolution. *Nature Plants*, **2021**, 7 (6)**:** 748-756. https://doi.org/10.1038/s41477-021-00933-x.
3. Fu F, Song C, Wen C, Yang L, Guo Y, Yang X, Shu Z, Li X, Feng Y, Liu B, et al. The *Metasequoia* genome and evolutionary relationships among redwoods. *Plant Communications*, **2023**, 4 (6)**:** 100643. https://doi.org/10.1016/j.xplc.2023.100643.
4. Scott AD, Zimin AV, Puiu D, Workman R, Britton M, Zaman S, Caballero M, Read AC, Bogdanove AJ, Burns E, et al. A Reference Genome Sequence for Giant Sequoia. *G3 Genes/Genomes/Genetics*, **2020**, 10 (11)**:** 3907-3919. https://doi.org/10.1534/g3.120.401612.
5. Neale DB, Zimin AV, Zaman S, Scott AD, Shrestha B, Workman RE, Puiu D, Allen BJ, Moore ZJ, Sekhwal MK, et al. Assembled and annotated 26.5 Gbp coast redwood genome: a resource for estimating evolutionary adaptive potential and investigating hexaploid origin. *G3 Genes/Genomes/Genetics*, **2021**, 12 (1)**:** https://doi.org/10.1093/g3journal/jkab380.
6. Cheng J, Wang X, Liu X, Zhu X, Li Z, Chu H, Wang Q, Lou Q, Cai B, Yang Y, et al. Chromosome-level genome of Himalayan yew provides insights into the origin and evolution of the paclitaxel biosynthetic pathway. *Mol Plant*, **2021**, 14 (7)**:** 1199-1209. https://doi.org/10.1016/j.molp.2021.04.015.
7. Song C, Fu F, Yang L, Niu Y, Tian Z, He X, Yang X, Chen J, Sun W, Wan T, et al. *Taxus yunnanensis* genome offers insights into gymnosperm phylogeny and taxol production. *Communications Biology*, **2021**, 4 (1)**:** 1203. https://doi.org/10.1038/s42003-021-02697-8.
8. Lou H, Song L, Li X, Zi H, Chen W, Gao Y, Zheng S, Fei Z, Sun X, Wu J. The *Torreya grandis* genome illuminates the origin and evolution of gymnosperm-specific sciadonic acid biosynthesis. *Nat Commun*, **2023**, 14 (1)**:** 1315. https://doi.org/10.1038/s41467-023-37038-2.
9. Niu S, Li J, Bo W, Yang W, Zuccolo A, Giacomello S, Chen X, Han F, Yang J, Song Y, et al. The Chinese pine genome and methylome unveil key features of conifer evolution. *Cell*, **2022**, 185 (1)**:** 204-217.e214. https://doi.org/10.1016/j.cell.2021.12.006.
10. Wan T, Liu Z, Leitch IJ, Xin H, Maggs-Kölling G, Gong Y, Li Z, Marais E, Liao Y, Dai C, et al. The *Welwitschia* genome reveals a unique biology underpinning extreme longevity in deserts. *Nat Commun*, **2021**, 12 (1)**:** 4247. https://doi.org/10.1038/s41467-021-24528-4.
11. Carey SB, Aközbek L, Lovell JT, Jenkins J, Healey AL, Shu S, Grabowski P, Yocca A, Stewart A, Jones T, et al. ZW sex chromosome structure in *Amborella trichopoda*. *Nature Plants*, **2024**, 10 (12)**:** 1944-1954. https://doi.org/10.1038/s41477-024-01858-x.
12. Zhang L, Chen F, Zhang X, Li Z, Zhao Y, Lohaus R, Chang X, Dong W, Ho SYW, Liu X, et al. The water lily genome and the early evolution of flowering plants. *Nature*, **2020**, 577 (7788)**:** 79-84. https://doi.org/10.1038/s41586-019-1852-5.
13. Tuskan GA, DiFazio S, Jansson S, Bohlmann J, Grigoriev I, Hellsten U, Putnam N, Ralph S, Rombauts S, Salamov A, et al. The Genome of Black Cottonwood, *Populus trichocarpa* (Torr. & Gray). *Science*, **2006**, 313 (5793)**:** 1596-1604. https://doi.org/10.1126/science.1128691.
14. Jaillon O, Aury J-M, Noel B, Policriti A, Clepet C, Casagrande A, Choisne N, Aubourg S, Vitulo N, Jubin C, et al. The grapevine genome sequence suggests ancestral hexaploidization in major angiosperm phyla. *Nature*, **2007**, 449 (7161)**:** 463-467. https://doi.org/10.1038/nature06148.

15. Ouyang S, Zhu W, Hamilton J, Lin H, Campbell M, Childs K, Thibaud-Nissen F, Malek RL, Lee Y, Zheng L, et al. The TIGR Rice Genome Annotation Resource: improvements and new features. *Nucleic Acids Res*, **2007**, 35**:** D883-D887. https://doi.org/10.1093/nar/gkl976.
16. Bornowski N, Michel KJ, Hamilton JP, Ou S, Seetharam AS, Jenkins J, Grimwood J, Plott C, Shu S, Talag J, et al. Genomic variation within the maize stiff-stalk heterotic germplasm pool. *The Plant Genome*, **2021**, 14 (3)**:** e20114. https://doi.org/10.1002/tpg2.20114.
